# Artery of Percheron Infarct: A Rare Case of Stroke in Pregnancy

**DOI:** 10.7759/cureus.30798

**Published:** 2022-10-28

**Authors:** Saliha M Abukhairat, Hadi S Alyami, Fatemah A Alshammaa, Fatema A Jamsheer, Nadeyah Y Baeyti, Maha M Aljehani, Mohd Al Ghadeeb

**Affiliations:** 1 General Practice, Jazan University, Jazan, SAU; 2 General Practice, King Faisal University, Hofuf, SAU; 3 General Practice, Arabian Gulf University, Manama, BHR; 4 General Practice, Royal College of Surgeons in Ireland, Manama, BHR; 5 General Practice, Ibn Sina National College for Medical Studies, Jeddah, SAU; 6 Radiology, King Fahad Hospital, Hofuf, SAU

**Keywords:** case report, computed tomography, magnetic resonance imaging, artery of percheron infract, pregnancy, stroke

## Abstract

Cerebrovascular complications, including strokes, are relatively frequent during pregnancy. Artery of Percheron is an anatomic variant that supplies the paramedian thalamus. Occlusion of this artery results in bilateral thalamic infarction, which manifests clinically with altered mental status. We present the case of a 33-year-old pregnant woman who presented with drowsiness and headache. The patient was at 12 weeks of gestation. She had normal vital signs, including blood pressure, and no focal neurological deficits were noted on physical examination. Magnetic resonance imaging of the brain was performed. The scan demonstrated abnormal high signal intensity in both thalami on T2-weighted images and corresponding restricted diffusion and low apparent diffusion coefficient. Such findings were consistent with the artery of Percheron infarct. The patient received antiplatelet therapy and exhibited gradual improvement. On discharge, the patient had a near-complete resolution of symptoms. Artery of Percheron infarct is a rare type of stroke. Clinicians should be able to recognize its clinical and imaging features.

## Introduction

Cardiovascular events are not uncommon during pregnancy. Certain physiological changes during pregnancy are associated with an increased risk of thromboembolic complications. These include alteration in the coagulation factors, cardiovascular hydrodynamics, and endothelial dysfunction [1]. It is estimated that the incidence of stroke in pregnant women is three times higher than in their non-pregnant counterparts [2]. Several conditions in pregnancy can predispose to strokes, such as eclampsia, posterior reversible encephalopathy syndrome, cerebral venous thrombosis, and HELLP syndrome [1]. Here, we present the case of a Percheron artery stroke in a pregnant woman, a rare type of stroke.

## Case presentation

We present the case of a 33-year-old pregnant woman who was brought to our emergency department by her husband who noticed her becoming very drowsy in the last few hours. The patient reported a history of worsening headaches and nausea for one week before the presentation. The patient had a history of hypertension and diabetes mellitus that was diagnosed at the age of 30 years. Her surgical history was remarkable for sleeve gastrectomy for weight control. Her medication history included metformin 500 mg and amlodipine 5 mg daily. The patient was pregnant at 12 weeks of gestation. There was no history of prior pregnancies or miscarriages. The family history was remarkable for migraine in her sisters. However, the patient did not experience any episodes of migraine. The patient worked as a school teacher. She never smoked or consumed alcohol.

On physical examination, the patient appeared drowsy. She was oriented to person, but not to time or place. The vital signs included a temperature of 37.1°C, a pulse rate of 82 beats per minute, a blood pressure of 105/72 mmHg, and a respiratory rate of 10 breaths per minute. There was no focal neurological deficit. A complete neurological assessment was not possible as the patient was not fully oriented. Laboratory parameters including hematological and biochemical parameters were within the normal reference range. These included the lipid profile and hemoglobin 1Ac.

**Figure 1:**
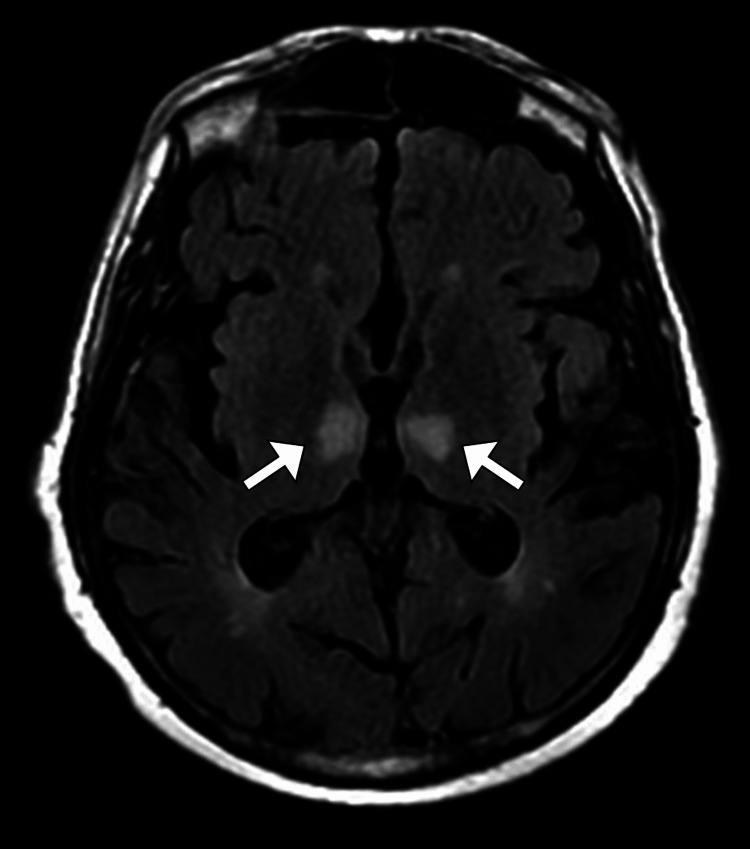
Axial FLAIR image of brain MRI shows abnormally increased signal intensity (arrows) in paramedian thalami. FLAIR: fluid-attenuated inversion recovery; MRI: magnetic resonance imaging

**Figure 2:**
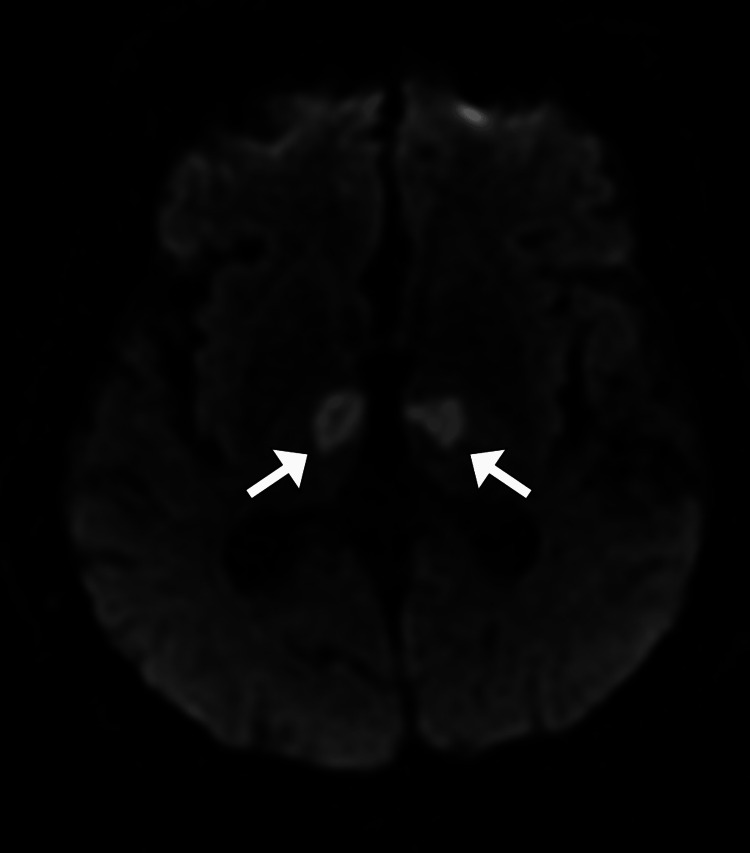
Axial DWI image of brain MRI shows areas of restricted diffusion (arrows) in paramedian thalami. DWI: diffusion-weighted imaging; MRI: magnetic resonance imaging

**Figure 3:**
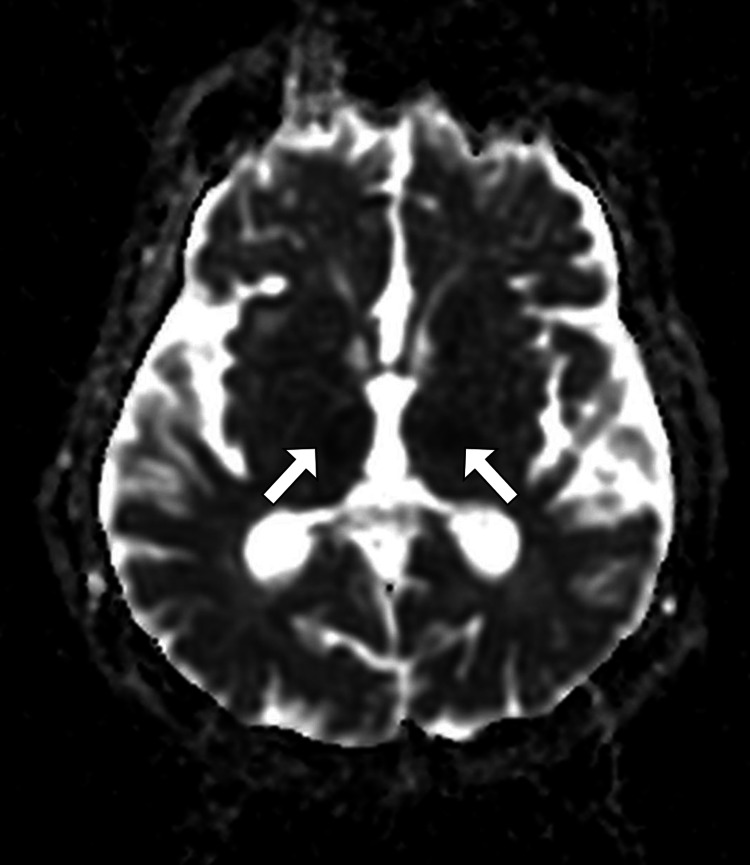
MRI ADC map image of the brain shows decreased diffusion in both thalami (arrows). MRI: magnetic resonance image; ADC: apparent diffusion coefficient

**Figure 4:**
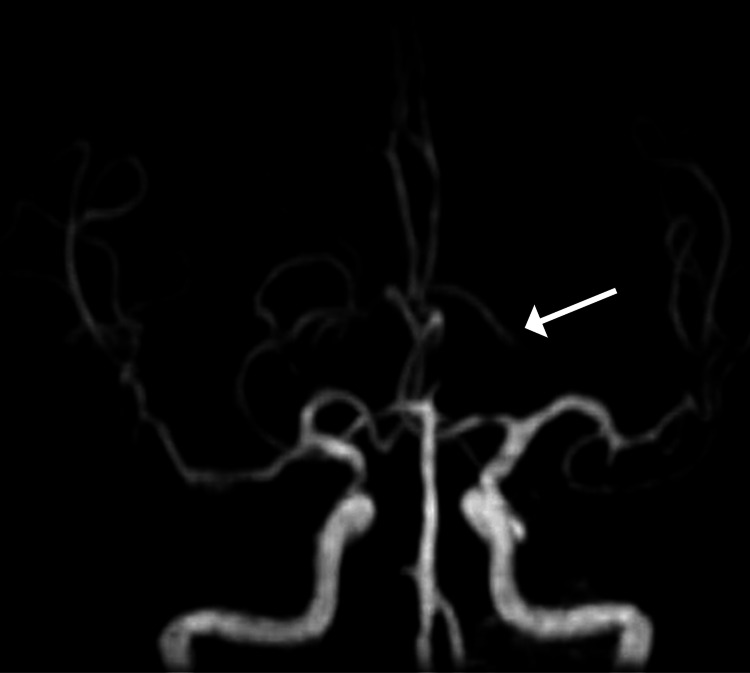
MRA image shows an abrupt cutoff of flow in an artery arising from the left posterior cerebral artery (arrow). However, there is no abnormal occlusion on the contralateral side. Hence, this finding suggests the diagnosis of an infarct due to the artery of Percheron. MRA: magnetic resonance angiography

The patient refused to have a computed tomography scan of the brain. Because the patient was pregnant and had sudden onset of decreased level of consciousness, magnetic resonance imaging of the brain was performed. The scan demonstrated foci of abnormally increased signal intensity in the medial margins of both thalami with associated restriction of diffusion and decreased apparent diffusion coefficient. These findings represented the diagnosis of acute infarction due to Percheron artery occlusion (Figures 1-4).

Subsequently, aspirin 81 mg was administered daily. The patient exhibited gradual improvement. She was discharged after one week of hospitalization. On discharge, the patient’s neurological deficits had resolved. No complications occurred throughout her remaining pregnancy period. The patient had an unremarkable spontaneous vaginal delivery at term. The complete thrombophilia workup revealed normal results. The thrombophilia workup included factor V Leiden, factor II mutation, homocysteine, protein C, protein S, and antithrombin III.

## Discussion

We report a rare case of artery of Percheron infarct. Four anatomic variants of the blood supply for the paramedian thalamus have been described. In type I, two paramedian arteries arise from the proximal segments of posterior cerebral arteries separately. In type IIa, two paramedian arteries arise from a single posterior cerebral artery. In type IIb, also known as the Percheron artery, a single paramedian artery arises from a single posterior cerebral artery and then bifurcates to supply both paramedian thalami. In type III, there is communication between both paramedian arteries that arise from both posterior cerebral arteries [3]. It is estimated that one-third of the population has an anatomic variant of the Percheron artery [3].

The occlusion of the Percheron artery results in an ischemic infarction of the bilateral paramedian thalami. The typical clinical manifestations of bilateral thalami infarcts include altered mental status, vertical gaze palsy, and memory impairments. The diagnosis of a Percheron stroke might be challenging, particularly in patients with normal findings on computed tomography [4]. Magnetic resonance imaging remains the diagnostic method of choice for an accurate and reliable diagnosis as a computed tomography scan has 50% sensitivity only [5]. Because the Percheron artery has a small caliber, its occlusion might not be demonstrated on computed tomography angiography or magnetic resonance imaging angiography [6]. In the present case, however, the occlusion of the artery of Percheron was evident on the magnetic resonance angiography. However, even with the absence of abnormalities on magnetic resonance angiography, the diagnosis of artery of Percheron should be considered, particularly in patients with altered sensorium and bilateral thalamic infarcts. The differential diagnosis of Percheron stroke is limited. For example, deep cerebral venous thrombosis can also present with headache, seizure, and neurological deficits with bilateral thalamic lesions on imaging. Wernicke’s encephalopathy and osmotic myelinolysis may share similar imaging features. However, their clinical presentations are different. Lastly, West Nile encephalitis and Japanese encephalitis may have similar clinical and imaging features to that of the artery of Percheron infarct. However, these infections typically occur in certain endemic areas [4,6].

The prognosis of the artery of Percheron stroke is favorable if the diagnosis is made rapidly within the thrombosis therapeutic window [4]. However, as in our case, the diagnosis is often delayed due to non-specific clinical manifestations of this type of stroke. Despite the fact that the patient did not receive thrombolysis therapy, the outcome in the present case was very favorable as a nearly complete resolution of symptoms occurred.

## Conclusions

The case demonstrated the clinical and imaging manifestations of the artery of Percheron stroke in a pregnant woman. Prompt diagnosis of this condition is facilitated by the awareness of the broad spectrum of stroke manifestations. Although it is a rare type of stroke, physicians should be familiar with the imaging features of Percheron stroke to initiate rapid treatment.
